# Supplementary material for: A Cancer-Specific Anti-Podocalyxin Monoclonal Antibody (humPcMab-60) Demonstrated Antitumor Efficacy in Pancreatic and Colorectal Cancer Xenograft Models
Source: Antibodies (Basel). 2025 Aug 11;14(3):67. doi: 10.3390/antib14030067 (PMC12372045; doi:10.3390/antib14030067)
Supplement: Supplementary file 1 [file antibodies-14-00067-s001.zip › antibodies-3758489-supplementary.pdf]

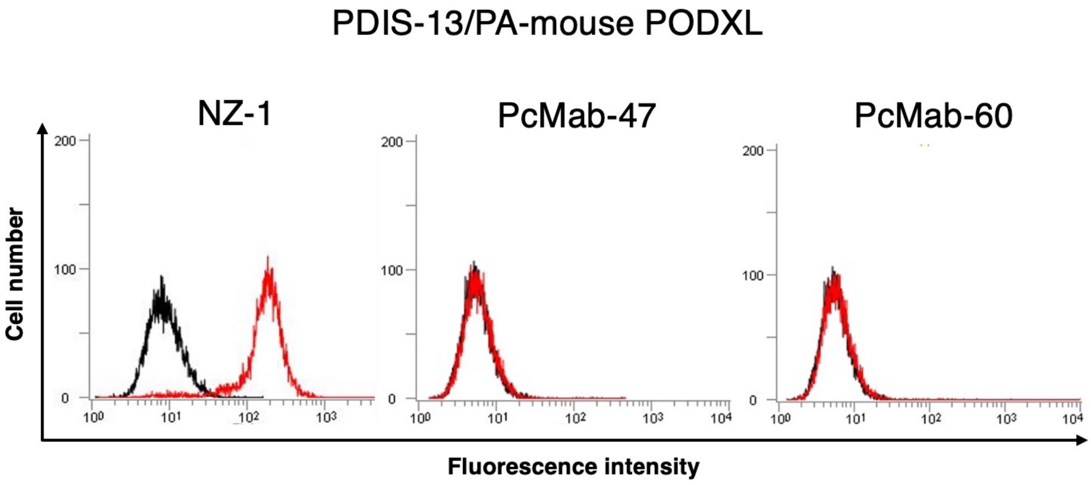

Supplementary figure S1. Flow cytometry analysis of PcMab-47 and PcMab-60 to PA-mouse PODXL-overexpressed PDIS-13 cells. The cells were treated with 10 μg/mL of PcMab-47 (red), 10 μg/mL of PcMab-60 (red), 1 μg/mL of NZ-1 (an anti-PA tag mAb, red), or blocking buffer (black). Then, the cells were treated with Alexa Fluor 488-conjugated anti-mouse IgG or Alexa Fluor 488-conjugated anti-rat IgG. Fluorescence data were analyzed using the SA3800 Cell Analyzer. PDIS-13, human PODXL-knockout LN229. Both PcMab-47 and PcMab-60 did not recognize mouse PODXL.
